# Discussions before the Kentucky State Dental Society

**Published:** 1878-07

**Authors:** F. Peabody


### Proceedings of Societies.

### DISCUSSIONS BEFORE THE KENTUCKY STATE DENTAL SOCIETY.

TUESDAY JUNE 4, 1878.

Pathology was the first subject taken up for discussion, to which Dr. A. O. Rawls spoke as follows:

I have but little to say on this subject, but by way of introduction I will present a branch of the subject a little different from anything given in the programme.

I refer to a disease that I have always considered one of the most difficult we have to contend with since it has been brought to our notice; one probably, which will require a great deal of time and much investigation, more than has ever been given to it, before we will be enabled to accomplish anything toward stopping its ravages.

I refer to that peculiar inflammatory action or diseased condition about the roots of the teeth and involving the periosteum, alveolus and gums. This affection is known by different names. I will not give it a name, unless I call it by the name given it by Dr. Rehwinkle, viz: "Pyorrhœa Alveolaris." I have attempted to bring this subject before the different associations, but without any definite results as to the proper mode of treatment of it, or its cause.

It seems to me our patients are losing more teeth, as the result of this disease, without the possibility of our helping them, than from any other cause. If it was something that we were becoming comparatively successful in combating, we might be encouraged; but we have thus far failed. Its effects are the same to-day as they were ten years ago, without a single case that we know of having been cured beyond the shadow of a doubt.

I know it is claimed by some that this disease is cured by local treatment, but they can not bring these cases before us, and prove that it is this disease, and prove that they have cured it. As a rule when this has been done they have shown to be cases of ordinary inflammatory action resulting from local interference, instead of cases after the nature of "Pyorrhœa Alveolaris." I have watched several cases very closely, and I have almost arrived at the conclusion that this disease is the result of undue use of mercury at some period or other in the life of the patient or the progenitors. There is almost always some show of mercury in the system, and a small quantity of mercury given to these patients usually aggravates the disease very perceptibly. It may be argued that it is the result of deposit of tartar or other irritating substances, but it has been proven time and again that the removal of all foreign matter does not accomplish any results except to ameliorate for a time the trouble, but does not permanently cure the disease. I have used with a few patients systemic treatment, and found them benefitted for a time just as they would have been in any similar disease. I have used medicines similar to those given for a mercurial taint, and have had results corresponding to those obtained by physicians under like circumstances, but I have not had time enough to carry this treatment sufficiently far to be enabled to express myself certainly on the subject.

We are aware that there is a vast difference between this disease as understood by a few eastern men and a few western men. I will do the western men the credit to say that I think they are right in their position. Some of the eastern men, and especially the advocates of Dr. Riggs' mode ot treatment of this disease, make it simply the ordinary inflammatory action, when in reality, there is no similarity whatever. If we can learn anything definite on this subject we will confer the greatest benefit on the profession that could possibly be conferred on this or any other association.

Dr. Taft—I apprehend that this particular disease to which Dr. Rawles has referred, in its frequency of occurrence as a systemic affection is overrated. I don't believe that there is very frequent occurrence of disease of the gums and alveolus in any shape, when all local causes are absent. We often overlook local as well as systemic causes. This term Pyorrhœa Alveolaris signifies simply a flow of pus from the alveolus. What are the conditions when there is this flow from the alvolus? Sometimes there is a wasting away of tissue both soft and hard. In other cases there will be for a long time some discharge of comparatively healthy pus without much or any wasting of the gums or alveolus.

The pabulum is perverted and transformed into pus instead of supplying the want of tissue. Then the gum is not nourished as it should be, and the circulation through it will always be more or less sluggish. There will be change, not necessarily tumefaction, but an apparent change in the gum. Sometimes this discharge of pus involves the loss of tissue—in many cases the loss of both soft and hard tissues. What is the cause of this discharge of pus? It is generally because the tissue does not make use of the nutrient material and so it is poured out, becomes degenerated, and assumes the form presented in the discharge. Sometimes the vital action throughout is of so low a character, that the system is not able to prepare the pabulum that should go to its nourishment. Then you know how it will suffer. This defective nutrition is oftentimes of a local character, that is, it is only in a limited territory. Now is such a local difficulty ever present without some local influence? I do not think these manifestations occur without local influence which occur either because of one or two reasons: either from a local active irritant or some undue enfeeblement of the part—one of the two and in many instances both. I apprehend that where there is trouble of this kind, resulting in absorption of the hard or soft tissues, that in nineteen cases out of twenty there is more or less active local irritants operating to produce it. My observation leads me to this conclusion: The mouth is a vulnerable point for abnormal conditions and processes. When a tooth is removed, absorption takes place to such an extent, sometimes as to very much weaken the support of the adjoining teeth, so that a tooth will be very poorly supported, and especially if in the articulation with the occluding teeth a tooth is forced towards the space from where the tooth was taken. In this way irritation is kept up all the while and the tendency to disease is produced by that cause alone, and it is not in the power of the dentist to prevent weakness and disease, unless the tooth is put in the condition it was before in respect to support.

The secretions of the mouth, when irritated, sometimes produce irritation of the gums. Improper use of the teeth sometimes occasion disease of the gums and alveolus.

I am not prepared to say, however, that irritation of the gums does not take place independent of any apparent local cause, but in the great majority of cases I have been able to find some local cause, which, when removed, the trouble is much mitigated or entirely removed. I have not seen a very large number of cases of diseased gums that could not be traced to some local affection. I should be pleased to examine such cases.

Dr. Herriot—Dr. Taft and Dr. Rawles are both right, looking at it from my stand point. I think it is local trouble caused by want of proper balance of the system, which is a systemic cause. I think it is very often caused by salivary calculus. I am willing to give Dr. Riggs the credit of the name of this disease, because he has given us the best instruments and best means of abating it, of any person in this country. My own teeth were diseased, and fifty dentists who examined them, said there was no salivary calculus. Then I went to Dr. Hunt, and he took a set of Riggs' instruments and spent about three hours in polishing my teeth to to get all the salivary calculus off; and to-day my teeth and gums are in better condition than they had been for months before. In nine cases out of ten, if you take the patient in time, this disease can be counteracted and cured by the removal of salivary calculus.

Dr. Hunt repoıted the case of Dr. Dickinson where one-third of the teeth was diseased, and it was cured by simply removing the calculus and polishing them up.

Dr. Peabody—There is perhaps no subject which we have under discussion more important than this, or about which we know less—in fact we don,t know anything about it. We do know, by observation and experience, that it is a very serious trouble. Dentists differ; some holding with Dr. Rawles that it is probably systemic, others with Dr. Taft that it is probably local. It may be that there are two diseases that have become mixed in the minds of dentists, or it is possible that they are so intimately connected that one follows the other. We want to get at the cause of this trouble, however, for we can treat no disease successfully until we know something about it. I am more than half inclined to believe myself that most of this serious trouble is what might be termed a blood disease, aggravated undoubtedly by local causes. Whether our attention had not been drawn to this disease in former years, or whether the disease has been very largely on the increase, is a matter for discussion; but we do know that we have more cases now, and I do believe that the disease is on the increase. I have seen many more cases in the last five years than previously. If the disease is on the increase, it can not be caused by local irritants, and what makes that more palpable is, that it comes where people take the most scrupulous care of their teeth. I have seen every tooth in the lower jaw perfectly sound, and every one in the upper jaw perfectly sound except one, and the gum would be puffed up and swollen, and soon followed by a smooth, glossy appearance. Take a probe and insert it along the posterior approximal surface and it will pass from one-third to one-half the length of the root, and in many instances you can't detect one particle of foreign deposit. Undoubtedly in many instances this disease is aggravated by local deposit, and in some instances local causes may produce an appearance of this disease, while in reality there is no disease at all. Dr. Peabody related the case of a right lateral incisor that had passed so far from the center as to lap over the other tooth. He brought it to its proper place by ligature and kept it there six months, but upon their removal at the end of that time the tooth went back. He then brought the tooth to position, inserted a gold bar into each cavity, and plugged it in. This was kept perfectly clean for two years, when it was removed the tooth again left its proper position, and eventually had to be extracted. In this case the physique of the patient was very slight, but in another case the patient weighed two hundred and fifty pounds, the teeth were perfectly clean and beautiful, but the gums became loose in the same way. In the latter case he concluded the roots were absorbed and extracted the teeth, when he found the roots just as clean as those of any perfectly sound tooth. He therefore believed the cause of this trouble to be in the blood; that it was not only systemic but hereditary. He had himself seen it in the same tooth, not extending from tooth to tooth, but confining itself to a particular tooth, in the mother, in the sister, in the child, and he did not see how local irritants could produce the disease in that peculiar way, and he could but think that the cause was in the blood and required systemic treatment to eradicate it. The treatment of Dr. Riggs had done a great deal of good, but he believed that the majority of the cases treated by Dr. Riggs had been the result of local causes and not the real disease.

Dr. Herriott—In those cases where there is no local irritant do you find the flow of pus equal to that where there is a local irritant?

Dr. Peabody—I think in the majority of cases it was, but in many instances there has been no appreciable flow of pus at all.

Dr. Rawles—I think Dr. Peabody has struck the key note to this trouble, but we must investigate further before we can hope to do anything with this disease. Dr. Taft was right in attributing much of the trouble to local irritants, and the condition of the system which brought it about; but we don't discriminate sufficiently between these diseases to understand how to treat them. What we want is to have the profession make an investigation so that we may know what to do. One reason I had for believing it a systemic disease, or poisoning of the circulating fluids of the body was, that wherever there is any degree of malaria, or where there has been mercurial treatment, the disease occurs with greater frequency than elsewhere. Cincinnati has very little malarial influences and that is probably the reason why Dr. Taft has not seen many cases of this kind, but let him go out on the Wabash and he will find this disease equal to one-half the diseases of the teeth, and that was why they were so unsuccessful in treating exposed pulps in that country. As Dr. Peabody said it is undoubtedly hereditary. Wherever I find these cases, the disease is in the family throughout, and it is undoubtedly on the increase. I think there should be some distinction between this disease and Riggs' disease. The one is systemic and the other local. He had found the disease in the healthiest patients.

Dr. Reid—Most of the success of Dr. Riggs' treatment is due to the time and care he bestows upon the teeth. If there is any one thing that is neglected by the profession it is in the matter of cleansing the teeth properly.

Dr. Rowdebush thought the disease hereditary to some extent at least. He had known the disease to proceed without any accompanying discharge of pus. Cited the case of a family where all the females had lost the teeth without any flow of pus. Had applied carbolic acid, white oak bark and other astringents without any permanent relief. Had never seen a case where the disease was arrested.

Dr. Kidd reported a case that had come under his observation, where the teeth had been lost, without decay or any flow of pus whatever.

Dr. McMillan—I think localities have a great deal to do with this disease. I have for three years made very close observation of the different sections of my country. We have a section called the Dry Ridge, where there is no malaria, and not one patient in fifty from that section is troubled with this disease, while from other sections where malarial influences exist I have a much greater number. I also have found patients with perfectly good teeth suffering from the disease. I find it largely on the increase. Cited the case of a lady with perfectly good teeth who suffered from the disease. He treated them with aromatic sulphuric acid, and afterward with calendula, until they were apparently well, but the patient afterwards returned with the disease in full possession of her teeth, just as when he commenced to treat them.

Dr. Taft said he had seen imperfection of tissue transmitted hereditarily. He saw no reason why a portion of bone, or even of soft tissue, might not be hereditarily defective, or more susceptible to disease than the tissues around it. There is not a hereditary transmission of disease, but a susceptibility to disease. As to the number of cases in one locality or one family, he would say that the same conditions existed in the many that did in the one, and so occasioned the same results. Necrosis of the margin of the alveolar process is found in some instances to be a source of irritation. The septum is more frequently affected than the lateral portions. The removal of this is often required before restoration in the parts about it can at all be restored.

Dr. Rawles—We have done that repeatedly; why don't it cure them?

Dr. Taft—I don't claim that it will cure always, but that is rational treatment so far as it goes. If the blood is impoverished it should be nourished. Give it the proper food. If it is poisoned, purify it, and give it good circulation through the effected parts, and then you have done all you can to effect a cure.

Dr. Herriot said that he and Dr. Rehwinkle had almost quarreled in a discussion of this subject, because they thought they disagreed in its treatment, whereas they found afterwards that they were talking about entirely different diseases, and the reason was, that Dr. Rhewinkle lived in a region where malaria was so bad that a man couldn't keep a saloon without keeping quinine cocktails, while he lived in a region where malaria was unknown.

Dr. Roudebush—What class of patients is the disease most confined to—plethoric or ænemic?

Dr. Peabody—I have had but very few plethoric patients, mostly ænemic. Subject passed.

At the close of the discussion other subjects were called in their regular order, but for various reasons were temporarily passed, and the subject of anæsthetics was taken up for discussion.

Dr. Canine, who was one of the committee appointed to write an essay on this subject, stated that he had not prepared a paper, but that he would say he found the use of gas constantly and largely increasing in his business; that he had given it to as many as twelve persons in one day, and had given it as high as six times to one patient, and never hesitated to do that if he thought the patient could bear it. Still from what he had heard from other dentists and from patients, he thought he had rather unusual success with gas. Since he began the use of nitrous oxide gas he had seen no alarming symptoms in his patients, but before that, when he used gas manufactured by himself, he had cases that very seriously alarmed him. However, he had never killed anybody with it, and believed it perfectly safe to administer to patients.

Dr. Kidd—How do you tell when the patient has enough?

Dr. Canine—That I can hardly tell. One of the principal things is the nervous twitching of the fingers. Another sign that they have taken enough is the fact that the last thing they do before losing consciousness is to swallow. Nothing is an infallible sign, however, and the dentist must be governed largely by his judgment in its administration. I generally give it according to the work I have to perform. If the amount of work to be performed is very great, I give a larger quantity of gas and *vice versa.*

Dr. Rawles—Did you ever see any ill effects some time after administering the gas ?

Dr. Canine—Not that could be distinctly traced to that.

Dr. Cassidy being called on to give his views on the subject, said: I believe, though it is only a theory of mine, that nitrous oxide gas is a supporter of combustion, and oxygen its only active agent. Taken into the lungs it supercedes the air, and the affinity between the nitrogen and the oxygen is so feeble that it is overcome, the oxygen attacks the hydrocarbon of the body as it naturally does, and that produces carbonic acid gas. Then that being more abundant than is natural, it takes possession of the nervous centers for the time being, but as soon as it is expelled, consciousness returns. Anæsthesia is due to the overabundance of carbonic acid gas—the same in effect as though the patient were suffocated for the time being, Anæsthesia is simply asphyxia to a certain extent. I think Dr. Canine will say that there is a greater quantity of carbonic acid gas exhaled than is natural. One reason for this increased amount of carbonic acid gas being generated in the body is the undue excitement of the patient. Get excited about anything and there is a greater amount of carbonic acid gas formed in the body. I administered gas regularly for four years, that is the old fashioned gas, but that is the same thing except that liquid nitrous oxide gas is more pure, and I consider the laughing gas dangerous in any case. I am not using gas now, chiefly because it was too much trouble to administer. I have never heard of any case that proved fatal except one in England, and that I think would have been fatal any way on account of the patient's heart. I do consider, however, that it is dangerous in some cases on account of the congestion produced by the rapid accumulation of carbonic acid gas. I think it about on the same principle as drowning a man to a certain extent, in order to bring him to a state of unconsciousness sufficient to operate on him. I never had any patients to die under my hands, though I have had some rather serious times in giving it. I believe seven teeth is the largest number I ever extracted at one administration, but on the other hand I have had to give it as many as three times for one tooth.

Dr. Herriot—In this report of Dr. Cassidy's I think perhaps he don't fully realize what he has been saying. He has uttered sentiments here that in my estimation are not true. The anæsthetic for a dentist to use is gas in its liquid form, not in the form in which he used it. I haven't found a single case yet in all my experience where liquid nitrous oxide gas has been faithfully administered that has proved fatal. There is not a single case on record so far as I have been able to discover, and I have taken particular pains to inform myself upon the subject. But I have found any amount of injurious effects from extracting teeth from nervous patients without administering any anæsthetic. Ether and chloroform are undoubtedly dangerous agents in the hands of dentists. He cited the case of a patient who had taken one hundred gallons of gas without any ill effects.

Dr. Roudebush thought that all anæsthetics should be handled with a great deal of care. The great wonder was that more patients did not die under their administration. He had never given nitrous oxide gas himself, but Dr. Dawson had told him that the only safety in the gas was that it passed off like a whiff. If its effects were as lasting as those of chloroform, it would kill everybody who took it. He himself gave chloroform in preference, and had given it for twenty-five years. He always did it, however, under the most favorable circumstances. He never covered up the face of the patients, as they did in administering gas, but gave them plenty of fresh air. He never gave it immediately after eating, and always had the clothes of the patient loose and comfortable; and though he had performed very extended operations, he had never seen any bad effects from the use of it.

Dr. Herriot—Do you know of a single case where death occurred from the use of nitrous oxide gas in its liquid form.

Dr. Roudebush—I can't give names or dates, but I remember hearing of one in this place, and also the case of a lady in Cumminsville, who turned spotted all over and became perfectly insane. I don't know, however, whether that was from gas in its liquid form.

Dr. Herriot referred the members of the association to a little book by Dr. Turnbull, of Philadelphia, for an interesting discussion of this question.

Dr. Canine—I think it is necessary, of course, to use care and judgment in the administration of nitrous oxide gas or any other anæsthetic. When a patient comes into my office and wants to take gas, I first ask, how long is it since you ate anything? If the answer is "three or four hours," I am satisfied as to that point. The next thing I want to know is whether there is any heart disease. If the reply is in the negative, that is satisfactory. If the patient is a lady, I then want to know if she is laced tightly or whether she can take a full, strong breath. If she can breathe freely, I request her to be seated, but instead of setting her up in an erect position, I incline her very far back, and then I go to work as though it were a life and death matter. I always feel every pulse from the time I begin to give the gas until I stop, and if any one says that the pulse runs down, he simply don't know anything about it. Then I talk to them gently and kindly during its administration, and try to convince them that it is not the dreadful operation they perhaps supposed it was, and if it is one who I think will become greatly excited, I kindly, but firmly give her to understand that I want no foolishness, and I never have any trouble, or find that it causes any bad effects. Cited the case of a lady partly insane, to whom he administered gas without any ill effects.

Dr. Goddard said he supposed he was the first man in the state of Kentucky who administered anæsthetics. He gave it first in the form of ether. He first experimented on a negro woman whom he fully expected to kill, but she survived. He came very nearly killing one woman, though so near, that it took her four or five hours to recover. He believed with Dr. Cassidy that anæsthesia is dangerous in any form except one, and that is to take two or three long breaths, the last one longest, and then the patients would not feel half the pain that they otherwise would. That is the anæsthetic that God gave us, and it is the only one that is not harmful. Adjourned.

The association convened at eight-thirty p. m., and resumed the discussion of anæsthetics as follows:

Dr. Peabody said that he kept liquid nitrous oxide gas and did administer it when his patients required it, but he never advised giving it, and never did give it unless his patients thought they could not get along without it. He had patients come to his office and tell him that Dr. so and so had given them gas about a year before, and they had never entirely recovered from the effects, and they didn't want any more gas. He thought the assertion of Dr. Cassidy, that it was dangerous was positively true—there was no anæsthetic in the world that was not more or less dangerous. He always used it with the greatest care and caution; always inquiring into the physical condition of the patient, etc., as described by Dr. Canine. He would not hesitate to make the unqualified assertion that nitrous oxide gas, ether and chloroform are liable to leave deleterious effects upon the system, even when administered with great care, and when given carelessly as so many of those who use it freely do give it, its consequences are unquestionably serious. He formerly administered ether very freely, and thought he would not exagerate if he said he had given it by the gallon daily, but he did not do so now. However, he considered ether much safer than chloroform, but not as safe as nitrous oxide gas, and even that does leave its bad effects on the system sometimes. Subject passed.

The next subject taken up was Chemistry, upon which Dr. Cassidy read a paper. It was well received and at its close Dr. Smith, of Cincinnati, said he desired to call attention to the explanation of Dr. Bridgman, of England, as to some of the various theories attempted to be explained by the disciples of the new departure. Mr. Bridgman claims that contact is not necessary in order to produce an electric current, which is in direct opposition to the theory held by Faraday and most other chemists. Mr. Bridgman immerses a copper wire in acid; it is not polarized, but immediately upon exposing any portion of it to the air, it become polarized and there is a rapid wasting away of the wire, accompanied by a deposit on the exposed end which shows there is an electric current. He makes the same experiment with zinc. Now if a tooth is filled, and a portion of it submerged, the portion not submerged will have polaric action opposite to the portion which is submerged, and hence at the poiut of contact, galvanic action. Dr. Flagg says they have never been able to show the electric current, but if a tooth is filled with amalgam, I can readily see from their standpoint, that you have a partial galvanic action there which might prevent decomposition of the tooth. I can't see, though, how you have electric action with out space, but the smaller the space that you have the more fatal its consequences. The wider the space the safer it is. This is an interesting subject to me, but like Dr. Flagg, "for the life of me I can't understand it." Cited the case of an operation where the filling in the dead teeth was perfect, while in the living teeth it was invariably imperfect, which seemed to disprove the theory that vitality was absolutely necessary for the preservation of the teeth.

Dr. Peabody—There is a certain class of teeth that the best operators in the world fail to save with gold filling. In many instances tin is a very much better preserver of dentine, from the action of conducting influences, whether electrical or acidulated; particularly in that class of frail white teeth, that are attacked more particularly by white decay.

Now I would like to ask Dr. Cassidy, what would be the effect of any base metal, tin or anything else, in regard to galvanic action, outside of its being a nonconductor.

Dr. Cassidy—If the oxide would be insoluble in a liquid it would arrest decomposition of course, and tin is insoluble in saliva, and is a nonconductor?

Dr. Peabody then cited the case of a lady who came to him and said that she had paid one of the most eminent operators in the country five hundred dollars to attend to her teeth and he had filled them with gold, but without satisfactory results. Dr. Peabody removed all the gold fillings, probably twenty in number, and filled the teeth with tin, and he thought he would be able to preserve them. He also related the case of a patient for whom he was filling a tooth, when the napkin became displaced, and the burnisher touched his lip, the patient immmediately winced. He tried it again with the same result. He then replaced the napkin and completed the burnishing without further pain. He at once concluded that there was an amalgam filling in the mouth, and although the patient declared there was not, he found, after examination that the second bicuspid contained an amalgam filling. He then touched this filling and the one he had just put in with the burnisher, and the patient jumped; but upon touching the gold fillings there was no response whatever. He removed the amalgam filling, then burnished the one he had just put in and obtained no response at all. He did not understand it then or now. Subject passed.

TO BE CONTINUED.

---

### MINUTES OF THE KENTUCKY STATE DENTAL ASSOCIATION.

HELD AT COVINGTON, JUNE 4TH, 5TH AND 6TH, 1878.

The eighth annual meeting of the Kentucky State Dental Association was held at the court house of the city of Covington, Tuesday, June 4th, 1878.

The association was called to order at 2:30 o'clock by the president, Dr. A. W. Smith, of Richmond, and Prof. J. Taft, of Cincinnati, being called on, opened the session with prayer.

The roll was called by the secretary, and the following officers and members answered to their names: A. W. Smith, president; C. G. Edwards, vice-president; F. Peabody, secretary; J. F. Canine, treasurer; Wm. H. Goddard, W. W. Justice, Jno. T. McMillan, executive committee; J. Hooper, board of censors; W. G. Redmon, C. E. Dunn, A. O. Rawles, J. B. Kidd, J. S. Cassidy, B. O. Doyle and J. Taft; H. M. Reid, H. A. Smith and W. M. Herriott, honorary members.

The minutes of the last annual meeting were read by the secretary and approved.

The president requested tnat the delivery of his address be deferred until the next day, which was granted.

On motion of Dr. Rawles, dentists and physicians feeling an interest in the meetings of the association were invited to attend and the courtesies of the floor extended to them.

Dr. Goddard called for the reading of a motion made by him last year in relation to changing article third of the constitution relating to time of electing officers. Motion put and carried.

On motion the hours for the meeting of the association were set as follows: morning session from 8:30 to 12; afternoon session from 2 to 6; evening session from 8 to 10.

The executive committee reported the operators appointed for clinics to be Drs. H. M. Reid, A. W. Smith, F. Peabody and B. O. Doyle.

A phonographer was employed at ten dollars a day to report the discussions.

The executive committee reported to having examined the accounts of the treasurer and found them to be correct. Report received.

On motion the regular order of business was suspended and miscellaneous business taken up.

On motion of Dr. Goddard, a committee of three was appointed to draft resolutions on the death of Prof. Geo. T. Barker, of Philadelphia. The committee as appointea by the chair was composed of Drs. Goddard, Taft and Peabody.

Dr. Rawles called for a report from the committee who had charge of the bill for the regulation of the practice of dentistry in the state. The chairman had no particular report to make, simply stating that the bill had been presented and passed, and is now a law of the state.

Dr. Goddard moved an amendment to article six of the by-laws, that it shall read, "the censors examining and executive committee," examining committee not now being in the law. Carried.

On motion, the Ohio Dental College was the place appointed for clinics, and the time the following Wednesday morning from eight to ten o'clock.

Adjourned till eight o'clock the following morning.

### SECOND DAY—WEDNESDAY MORNING.

The morning session was devoted to clinics held at the dental college in Cincinnati. Operations were performed by Drs. Reid, Doyle and Peabody. Dr. Peabody also demonstrated the filling of roots of teeth with lead.

### WEDNESDAY AFTERNOON.

The association was called to order by the president, Dr. A. W. Smith, in the court house at Covington. The roll call and reading the minutes were dispensed with.

The resolution offered by Dr. Goddard the preceding day on the amendment of the sixth article of the by-laws was voted on and adopted.

The president then read his address which was received with applause and referred to a committee of three to select such portions as it was deemed advisable for the association to take action on, and present them to the association. Committee, Drs. Hooper, Dunn and Justice.

The first subject on the programme for discussion, anatomy, embracing the following questions: Do the nerves of extracted teeth unite in replanting when in a healthy condition? What is the condition of the alveolar process when there is abscess on the tooth? was then taken up. The committee on that subject not being present it was passed and the next subject, physiology, including the provisions of nature for the protection of exposed pulps and the circulation of pulp vessels, was introduced. No paper being presented the subject was temporarily passed, and pathology, with the following points, secondary dentine, osseous pulps, abnormal secretions, exostosis, action and effects of dead pulps, was presented. The subject was not represented by any paper, but was discussed by Drs. Rawles, Taft, Peabody, Herriott, Kidd and McMillan. The discussion was prolonged and of more than ordinary interest. The fourth subject, chemistry; the different agents that act to produce decay or disintegration of dentine and enamel, and their modes of action, with the other subjects for discussion was laid over. The following resolution was offered by Dr. Goddard: *Resolved*, That the duties of the examining committee shall be to carry out fully the requirements of the law passed by the legislature in regard to practitioners of our specialty in the state, and to register in a book provided for that purpose, in alphabetical order, every one (if possible) who was engaged in the practice of dentistry at the time of the passage of the bill. The resolution was voted on and carried. Dr. Herriott, of Indianapolis, extended a cordial invitation from the Indiana Association of Dentists to the Kentucky Dental Association to meet with them at Indianapolis at their next meeting to be held June 25th. The association returned its thanks for the courtesy, and hoped as many of its members as possible would attend. The subject of anæsthetics was then called up. There was no paper, but the subject was discussed by Drs. Canine, Rawles, Cassidy and Peabody. Dr. Cassidy gave his theory of the action of nitrous oxide gas, and explained that carbonic acid gas was the probable agent in producing anæsthesia and asphyxia.

Adjourned at six o'clock.

WEDNESDAY NIGHT SESSION.

The association was called to order by the president at a quarter past eight. The subject of anæsthesia not being closed was taken up and discussed by Dr. Peabody, then passed. Chemistry was next in order on the programme. An able and very interesting paper was read by Prof. J. S. Cassidy on oral electricity. This subject, in connection with operative dentistry was discussed by Drs. H. A. Smith and F. Peabody. This subject was then closed and the subject of hygiene came before the association. With this was general prophylactic treatment; use of phosphates and the results; care of the teeth in general. No paper was presented on this subject and no discussion. Therapeutics was next in order; particular treatment of neuralgia; action of therapeutic agents. On this subject Dr. McMillan read a very interesting paper. Drs. Taft, Roudebash and Sage discussed the subject. This was passed and Dr. A. Wilkes Smith, the president, addressed the association, and offered the following resolution:

*Resolved*, Whereas, Dr. George W. Priest, a former member of this association and a practicing dentist of the city of Louisville, was honored by being elected one term as secretary, and one term as president of this association, and whereas, the said George W. Priest has brought dishonor upon himself and discredit on this association in his resorting to public and unprofessional advertisements and practices, and whereas, there are members of this association holding diplomas with the name of said Priest signed to them as secretary and president, therefore be it

*Resolved*, That the diplomos held by said members in good standing at the present time, be and are hereby rescinded, and that said members be furnished with new diplomas with the name of some past honorable officers of this association on the same. Adopted.

On motion of Dr. Goddard, the incoming president was authorized to procure as many new diplomas as might be required to take the place of those signed by Dr. Priest. Carried. Adjourned to Thursday morning.

THURSDAY JUNE 6TH—MORNING SESSION.

The reading of the minutes was dispensed with, and Dr. Goddard read the following resolution of regret and sympathy:

*Whereas*, The members of the Kentucky State Dental Association have learned with deep regret the death on January 10th of Prof. Geo. T. Barker, of Philadelphia, intimately known to many of us personally, and to all professionally as a teacher and ardent co-worker in all that pertains to dental science. One who was ready to impart to others the knowledge he possessed—never withheld a kind and genial answer to any question asked him, his aim being to elevate all to his standard of professional excellence, and whose death is a loss to all interested in the advancement of our profession. We can not refrain from expressing how deeply we lament his untimely death. Therefore be it

*Resolved*, That the members of this association deeply sympathize with his colleagues of the dental institution with which he was connected, the many warm friends who were so devotedly attached to him and to the members of his afflicted family whose loss is irreparable.

*Resolved*, that a copy of the above preamble be sent under the seal of the association to the college of which he was late professor and to his afflicted family.

WM. H. GODDARD,
J. TAFT,
F. PEABODY.

The subject of surgery next came up. Dr. Edwards read an interesting paper which was discussed by Drs. Taft, A. W. Smith, F. Peabody, A. O. Rawles, McMillan and Leslie. On motion this subject was passed and the treatment of children's teeth taken up. A paper read by Dr. Kidd was discussed by Drs. Doyle and Goddard. On motion of Dr. Rawles this subject was passed temporarily to hear a voluntary essay by Dr. Peabody; after the reading of the paper a vote of thanks was passed to Dr. Peabody for his essay, and his labors in preparing and submitting his method of inserting pivot teeth.

The committee on the president's address made a report, and suggested that the matter of dental hygiene as spoken of in the paper be referred to the president A. W. Smith to prepare a hand book on the subject. Adjourned until two o'clock.

AFTERNOON SESSION JUNE 6TH.

The association met at two o'clock and was called to order by the president. The subject of Dr. Peabody's paper being up and no remarks being made as the subject was a new one, the treatment of children's teeth was again referred to, and remarks made by Drs. Goddard, Smith, Redman, Taft and Edwards.

Dr. Rawles moved to proceed to the election of officers. Carried. The election resulted as follows:

President, F. Peabody, Louisville; Vice-President, W. W. Justice, Winchester; Secretary, J. S. Cassidy, Covington; Treasurer, J. F. Canine, Louisville. Board of Censors, Jno. T. McMillan, Paris.

The President and Secretary are members of the examining committee ex-officio. The President being chairman.

Examining Committee, A. O. Rawles, Lexington three years; C. E. Dunn, Louisville two years; A. W. Smith, Richmond one year.

Executive Committee, Wm. H. Goddard, Louisville.

Louisville was selected as the place for the next annual meeting.

Delegates to the American Association were appointed as follows: Drs. Peabody, Hooper, Justice, Cassidy and Edwards.

A vote of thanks was extended to the mayor and common council of Covington for the use of the Court House.

Five dollars was appropriated to the janitor.

The thanks of the association were extended to Dr. J. S. Cassidy for his courtesies to the members of the association.

The secretary and vice-presidents names were substituted for that of Dr. Priest on diplomas.

On motion the secretary was ordered to issue a circular to all members of the profession in the state, requesting them to forward their name and residence to the secretary, to be placed in the book of record of those dentists practicing at the time of the passage of the law to regulate the practice of dentistry of the state. It is ordered that any dentist failing to respond to the circular will not have his name recorded, and may be liable to be proceeded against as not having been in practice at that time.

The executive committee submitted the following subjects for discussion at the next annual meeting, to which the president appointed the following committees.

Anatomy.—Do the nerves of extracted teeth unite in replanting? What are the conditions of the alveolar process when there is abscess on the teeth? C. G. Edwards, A. W. Smith.

Physiology.—The provisions of nature for the protection of exposed pulps. J. Hooper, J. B. Kidd.

Pathology.—Secondary Dentine, Osseous Pulps, Exostosis, Action and effect of dead teeth, A. W. Rawles, W. W. Justice.

Chemistry.—The different agents that act to produce decay or disintegration of dentine and enamel, and their modes of action. J. S. Cassidy, B. O. Doyle.

THERAPEUTICS.—Action of remedial agents upon diseased conditions causing absorption of the alveolar process and gums, vitiated conditions of the oral secretions, resulting from mercurial taints. C. E. Dunn, W. G. Redman.

OPERATIVE DENTISTRY.—The so-called new departure. S. Driggs, J. T. McMillan, Wm. H. Goddard.

VOLUNTARY ESSAYS.

MECHANICAL DENTISTRY.—J. F. Canine, L. W. Wells.

HISTOLOGY.—J. Taft, H. A. Smith.

After the installing of the officers, the association adjourned to meet in the city of Louisville the first Tuesday in June, 1879 F. PEABODY, Secretary.

