# Erratum

**Published:** 1878-08

**Authors:** 


### ERRATUM.

In July number, page 297 in second line of fourth paragraph for "tin," read "oxide of tin."

